# Brown tumor due to primary hyperparathyroidism in a familial case: a case report

**DOI:** 10.1186/s12902-023-01475-3

**Published:** 2023-10-08

**Authors:** Zongping Diao, Jianquan Zhang, Jiaqi Zhao, Weihu Sun, Zhengguo Pu

**Affiliations:** 1 Department of Ultrasound, Ruijin Hospital, Shanghai Jiaotong University School of Medicine, Shanghai, 200025 China; 2 Department of Interventional Ultrasound, Shanghai International Medical Center, Shanghai, 201318 China; 3 Department of Ultrasound, The Second Affiliated Hospital of Naval Medical University (Second Military Medical University), Shanghai, 200003 China; 4 Department of Ultrasound, Shanghai Fourth People’s Hospital Affiliated to Tongji University, Shanghai, 200434 China; 5 Department of Radiology, The Second Affiliated Hospital of Naval Medical University (Second Military Medical University), Shanghai, 200003 China

**Keywords:** Primary hyperparathyroidism, Familial disease, Brown tumor, Ectopic parathyroid adenoma, Case report

## Abstract

**Background:**

Primary hyperparathyroidism (PHPT) is an uncommon disorder characterised by hypercalcemia with an increased parathyroid hormone level. We reported a PHPT familial case with two subjects, a father and a daughter, and both of them had suffered from the brown tumor.

**Case presentation:**

The proband, a 43-year-old patient, developed parathyroid adenomas at the age of 15; a histologically confirmed right parathyroid adenoma was removed by parathyroidectomy; and after six months follow-up, the serum calcium level was normalised. At the age of thirty-three, a CT scan of his head and neck revealed a mass in the right maxilla, as well as PHPT (i.e., left inferior parathyroid adenoma). Then, he underwent a biopsy of an exophytic lesion in the right maxilla and was diagnosed by pathology as a brown tumor, with the serum calcium and PTH levels at 2.78 mmol/L and 221 pg/mL, respectively. Subsequently, the patient took a left inferior parathyroid microwave ablation with ultrasound guidance. After three months of follow-up, the serum calcium and PTH levels returned to normal, and the brown tumor was resolved. After three years, it mineralised as revealed in a CT scan. By the time he was 43 years old, during the 28-year follow-up period, the serum calcium and PTH levels were still within the normal range, and there was no discomfort reported. He has consistently taken calcium supplements throughout the 28 years. Since the initial diagnosis, his blood indicators of kidney function have been normal, and ultrasound showed renal calculus in the right kidney and a normal left kidney. The proband’s daughter, a 15-year-old girl, experienced left upper extremity pain for ten months. CT scan revealed a mass in the distal left radius, and a giant cell tumor was suspected. A surgical internal fixation was performed, and the pathology showed a brown tumor. Laboratory tests revealed a serum parathyroid hormone (PTH) level of 1554pg/mL, calcium level of 3.14 mmol/L, phosphorus level of 0.72 mmol/L, and alkaline phosphatase level of 1892 U/L. Given the osteitic changes and elevated levels of calcium and PTH, ultrasonography was performed, after which a mass was detected measuring 19 × 9 × 7 mm mixed with solid components and cystic fluid in the right thyroid gland. The results of ^99m^Tc-MIBI scintigraphy confirmed the abnormal accumulation of ^99m^Tc-MIBI in the right thyroid gland but not seen in the bilateral parathyroid glands. The patient underwent thyroidectomy, and the postoperative pathology report indicated an intra-thyroid ectopic parathyroid adenoma. The serum calcium and PTH levels became normal at 4 h after surgery. One to three months after operation, the serum calcium level was low, while the serum PTH level was high. Then, the patient was advised to take calcium supplements. Until the sixth month after the operation, the serum calcium level and serum PTH level returned to normal, and the bone pain was relieved. The patient’s blood tests for kidney function remained normal. There was no evidence of bilateral kidney disease (such as nephrolithiasis or nephrocalcinosis) detected by ultrasound scan. There were several similarities in the state of illness between these two subjects. Both the father and the daughter developed parathyroid adenomas at the age of 15, and there was no lesion in other endocrine glands. And genetic testing revealed mutations in the CDC73 genes in both father and daughter. On the other hand, there were also a few differences. The father’s first signs of brown tumor were in the right maxilla, while the daughter’s appeared in the distal left radius. The father presented pathological changes in the left and right parathyroid glands, whereas the daughter presented with an ectopic parathyroid adenoma in the right thyroid gland.

**Conclusion:**

We report a familial case in which father and daughter were diagnosed to have brown tumors due to parathyroid adenoma and ectopic parathyroid adenoma, and genetic testing revealed CDC73 gene mutations in both. Therefore, in the diagnostic and differential process of young patients having bone disease, clinicians should not only focus on the clinical manifestations of the skeleton, but also implement a comprehensive analysis of systemic symptoms, considering the possibility that the patient has familial PHPT.

## Background

Primary hyperparathyroidism (PHPT) is a sporadic disease that is mainly manifested in individuals aged 50–60 years [1], with a prevalence rate of 1–4 per 1,000. PHPT is identified as a familial disease in less than 10% of the total cases [2]. Genetic mutations, on the other hand, are present in 24–46% of PHPT cases in children [3]. Therefore, the possibility of familial inheritance should be considered in children with PHPT. Commonly identified hereditary syndromes associated with PHPT encompass multiple endocrine neoplasia (MEN) types 1, 2a, and 4, together with CASR or CDC73-related disorders [4]. Patients with overt PHPT have an increasing risk of neurocognitive dysfunction, cardiovascular morbidity and mortality [5]. The various familial PHPT syndromes have a wide range of clinical presentations at different stages. Thus, it is necessary for accurate classification to take into account an appropriate combination of genetic and pathological features, biochemical, clinical, radiological, and intraoperative findings when making treatment decisions [6]. In practice, however, it may be challenging for clinicians to immediately think of the possibility of familial PHPT when encountering cases of children with skeletal or renal symptoms. We report here a rare familial case involving a father and daughter who developed brown tumors in different locations.

## Case 1

The proband, a 43-year-old patient, was admitted to the hospital at the age of 15 (his symptoms first appeared in the 1990s) because of severe numbness in his face and extremities. A laboratory test revealed a serum calcium level of 3.6 mmol/L (normal range: 2.03–2.67 mmol/L), and a computed tomography (CT) scan showed a nodule in the right parathyroid gland. After performing parathyroidectomy on the patient, the lesion disappeared. The patient was then advised to take calcium supplements. After six months, the serum calcium level returned to normal.

At the age of 33 (18 years after parathyroidectomy), the patient complained of oropharyngeal discomfort and numbness/weakness in the extremities. Blood calcium and PTH levels at that time were measured to be 2.78 mmol/L (normal range: 2.11–2.52 mmol/L) and 221 pg/mL (normal range: 10–65 pg/mL), respectively. A CT scan results reported a mass in the right maxilla and an occupancy of the left inferior parathyroid region (possible parathyroid adenoma was considered). He underwent a biopsy of an exophytic lesion in the right maxilla, and the cytology result showed multiple multinucleated giant cells in the stroma with fibroblast infiltration and interstitial haemorrhage, diagnosed as a brown tumor. The patient then took the treatment of ultrasound-guided microwave ablation of the left inferior parathyroid gland. Three months after surgery, serum calcium and PTH levels became normal, and the brown tumor disappeared. Three years after surgery, it mineralised, as shown by a CT scan.

At the age of 43 (28 years after parathyroidectomy), as his daughter developed symptoms similar to those in his youth, the father visited a hospital again for medical examinations despite the absence of discomfort. The serum calcium level was 2.21 mmol/L, and the PTH level was 94 pg/mL. The results of scintigraphy, with technetium-99 methoxy-isobutyl-isonitrile (^99m^Tc-MIBI) as the scanning agent, confirmed the absence of tumor in the bilateral parathyroid glands. He has taken calcium supplements consistently for 28 years. Since his illness, blood indicators of kidney function have been normal, and ultrasound showed renal calculus in the right kidney and a normal left kidney. After collecting information about his family history, we learned that his mother was a radio announcer who died of unknown causes at the age of 70, and his father was a teacher who died of hypertension, while there have been no unusual symptoms observed in his sister until present.

## Case 2

**Fig. 1:**
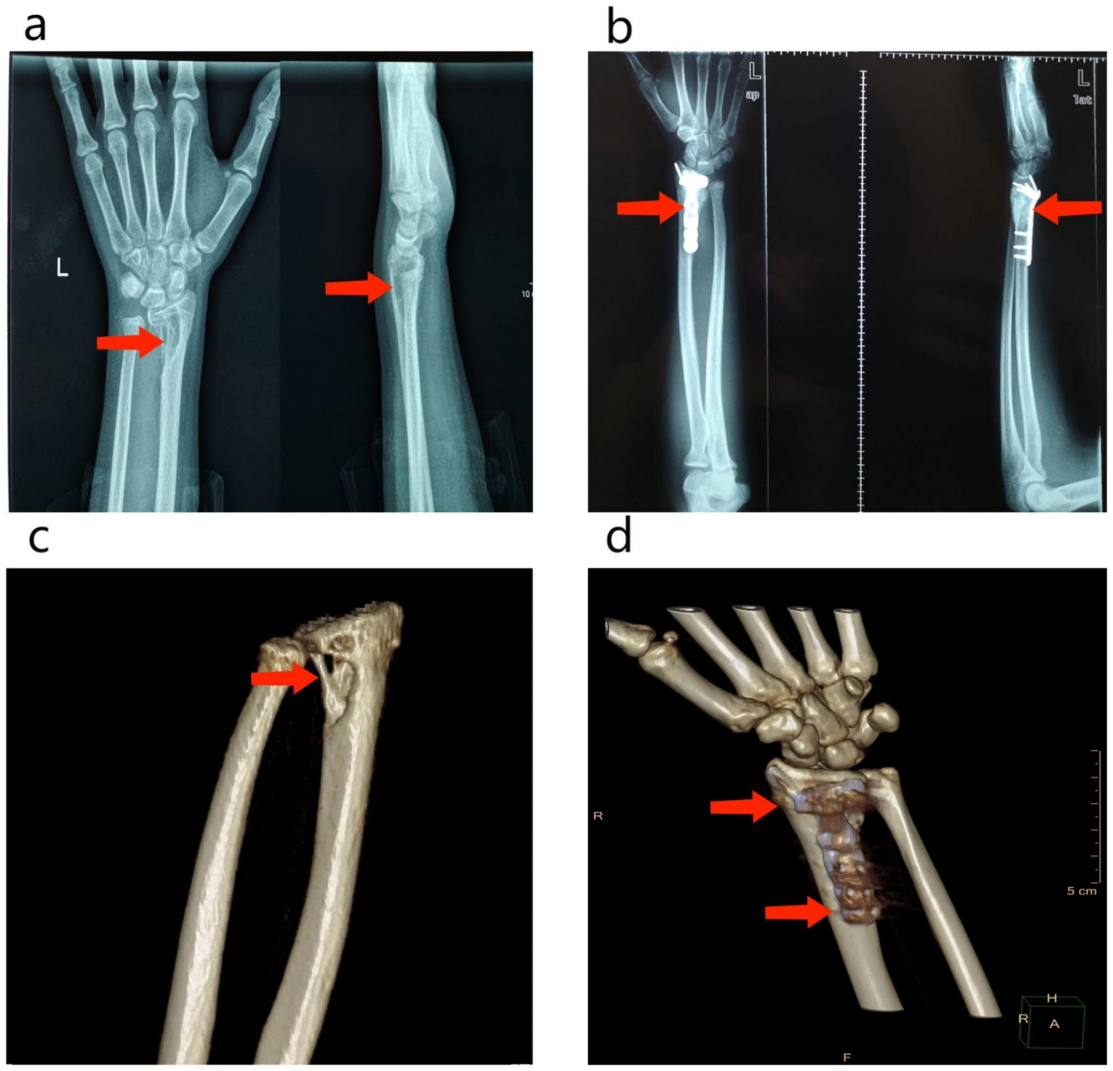
Before surgery **(a, c)** and three months after surgery **(b, d)**, the image of the left forearm shows the bone of the distal radius with cystic lesion **(a, c)**, as well as the image three months after surgery: union of the left distal radius after fixation with a long nail **(b, d)** (Case 2)

**Fig. 2:**
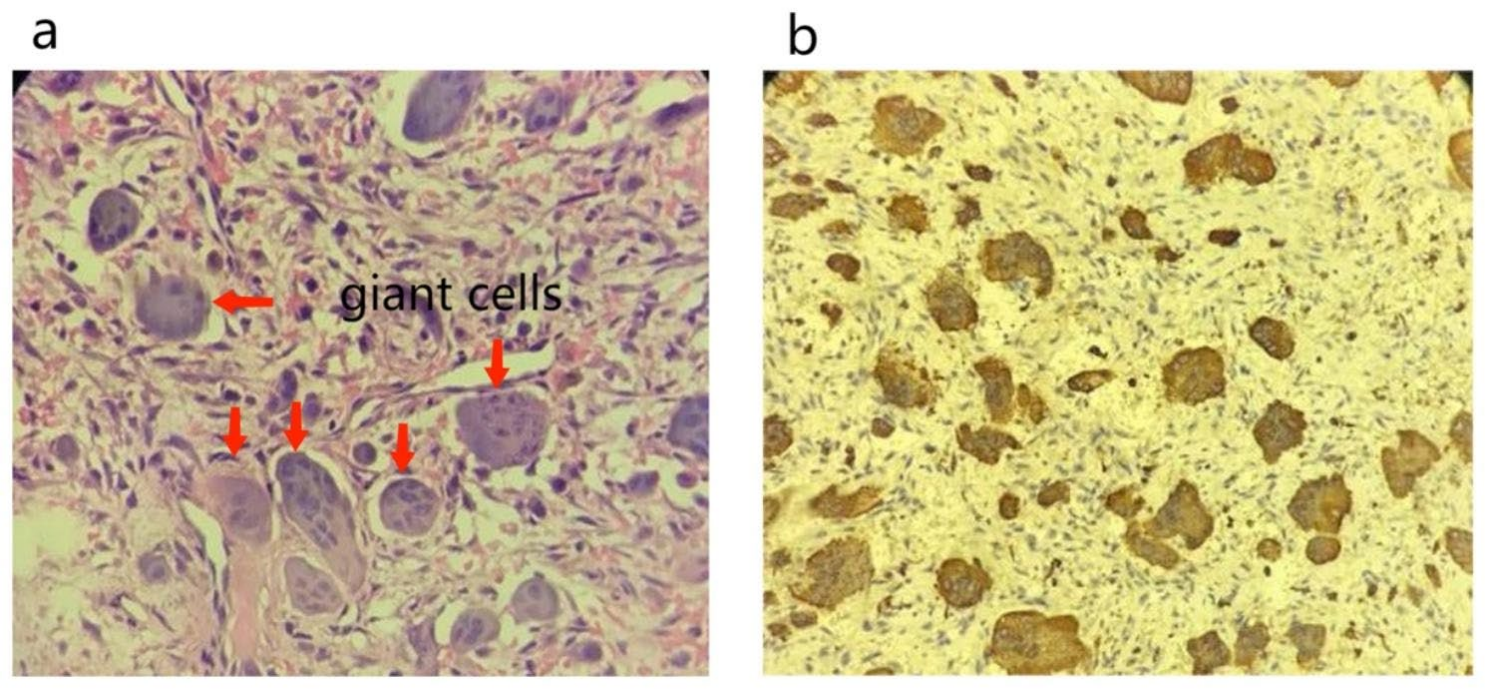
Histopathological examination report: photomicrograph of the left radius lesion, showing sheets of mononuclear cells admixed with multinucleated giant cells **(a)**, Hematoxylin and eosin stain (magnification ×100); Immunohistology, positive expression of CD68 (magnification ×100) **(b)**. (Case 2)

The proband’s daughter, a 15-year-old girl, experienced left upper extremity pain for ten months. She was diagnosed with a bone tumor in a hospital in her hometown and referred to our hospital. The X-ray scan revealed a mass in the distal left radius, and a giant cell tumor was suspected (Fig. 1). The patient underwent surgical fixation, and cytology examination indicated a brown tumor (Fig. 2).

**Fig. 3:**
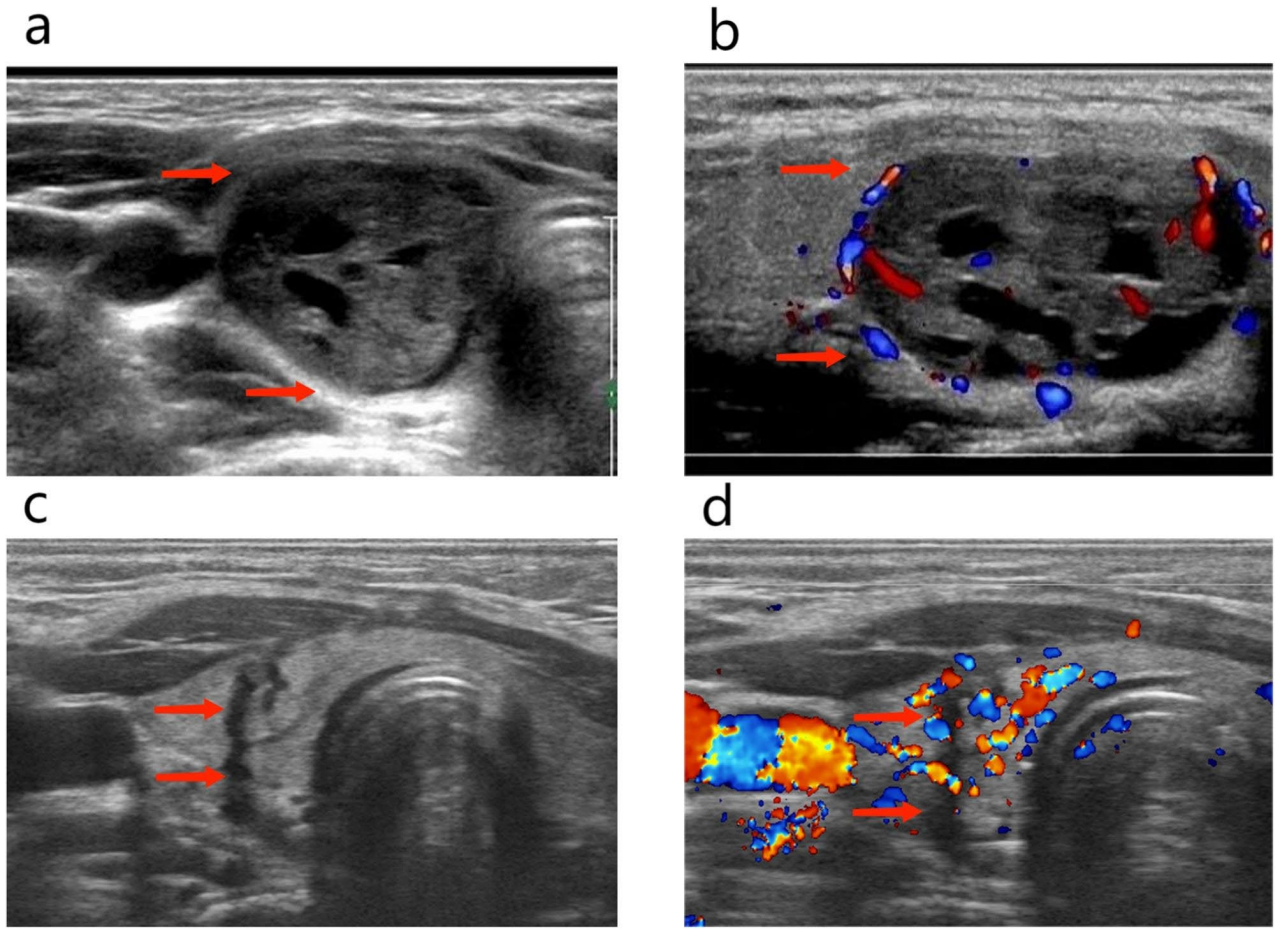
Before parathyroidectomy **(a, b)** and three months after parathyroidectomy **(c, d)**: ectopic parathyroid mass located in the parenchyma of the right lobe of the thyroid (red arrows), the image of the right thyroid three months after parathyroidectomy, the scar after the removal can be seen (red arrows). (Case 2)

**Fig. 4:**
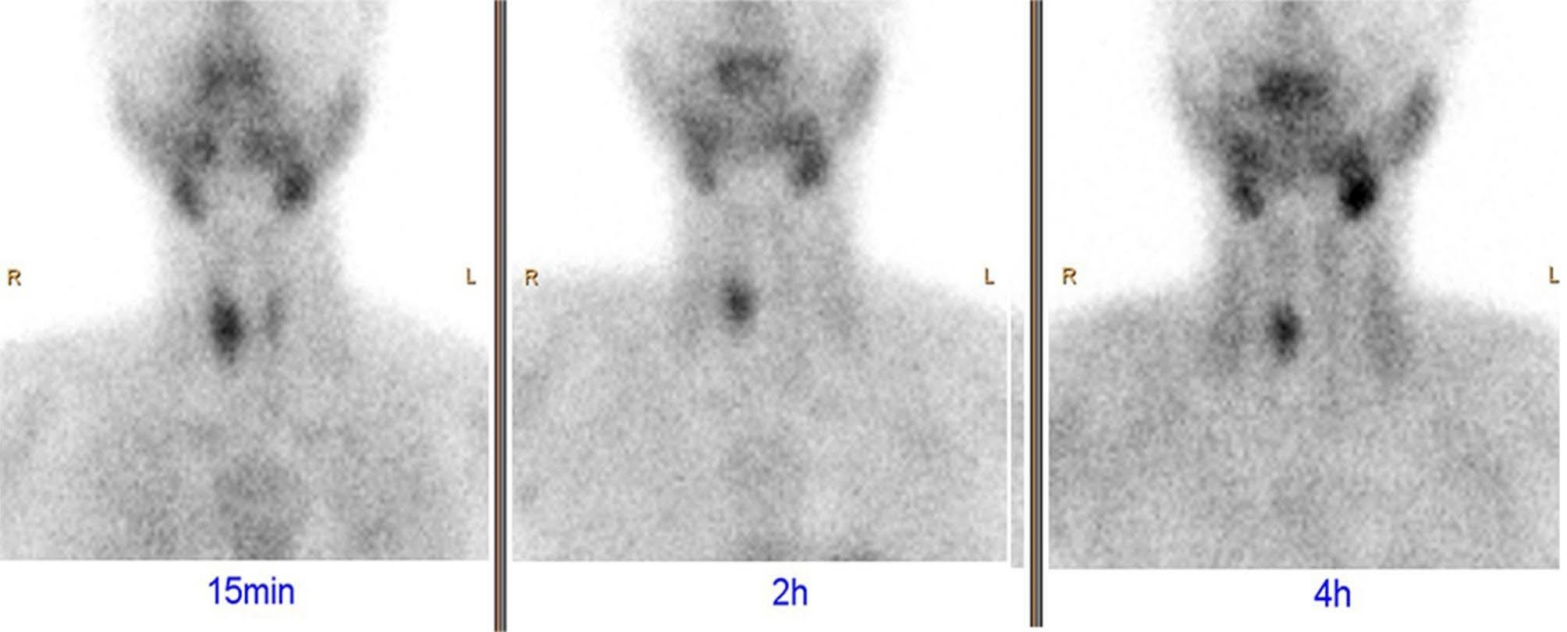
^99m^Tc-MIBI planar image shows relatively high activity in the lower part of the right lobe of the thyroid gland on 15 min and remains high concentration on the delayed image (2 h, 4 h). (Case 2)

On physical examination, the patient was slender. There were no developmental anomalies or deformities, and nor was there a palpable mass in the neck. Laboratory tests revealed a serum parathyroid hormone (PTH) level of 1554 pg/mL, a calcium level of 3.14 mmol/L, a phosphorus level of 0.72 mmol/L (normal range: 0.81–1.45 mmol/L), an albumin level of 45.4 g/L (normal range: 35–55 g/L), and an alkaline phosphatase level of 1892 U/L (normal range: 16–112 U/L). Because of the osteitis changes and the elevated calcium and PTH levels, she underwent ultrasonography. The results indicated the presence of a 19 × 9 × 7 mm mass mixed with solid components and cystic fluid in the right thyroid gland, which was identified as an intra-thyroid ectopic parathyroid adenoma (Fig. 3). The results of 99mTc-MIBI scintigraphy confirmed the abnormal accumulation of 99mTc-MIBI in the right thyroid but not in the bilateral parathyroids (Fig. 4).

After consultation with a multidisciplinary team comprising specialists from orthopaedic oncology, head and neck surgery, radiology and endocrinology, the patient underwent thyroidectomy and surgical fixation of the left radius. The postoperative pathology report showed an intra-thyroid ectopic parathyroid adenoma. The serum calcium and PTH levels normalised four hours after surgery. However, hypocalcemia occurred in the first month after surgery, with a calcium level of 2.05 mmol/L, and the patient was advised to take calcium supplements. During the three-month postoperative period, the serum calcium level remained relatively low, but the serum PTH level displayed a high level. By six months after surgery, the serum calcium level and the serum PTH level returned to normal levels, and the previously experienced bone pain was resolved. The trend of the blood indices before and after parathyroidectomy is shown in Table 1. The patient’s blood tests for kidney function remained within the normal range. The ultrasound scan showed no evidence of bilateral kidney disease (such as nephrolithiasis or nephrocalcinosis).

**Table 1:** Trend of blood tests before and after the parathyroidectomy of Case 2

Test	Reference range	Before-Parathyroidtomy	Post-Parathyroidtomy Hour 1	Post-Parathyroidtomy Day 30	Post-Parathyroidtomy Day 90	Post-Parathyroidtomy Day 180
PTH (pg/ml)	15–65	1554	26.15	298	187	18
Serum calci- um(mmol/L)	2.1–2.55	3.14	2.24	2.05	2.00	2.33
Phosphorus (mmol/L)	0.81–1.45	0.72	0.99	1.04	1.40	1.32
TSH (mU/L)	2.0–10.0	3.56			3.94	
TT3 (nmol/L)	1.54–3.08	1.98			2.03	
TT4 (ug/dl)	4.5–12	6.09			4.27	

There are four notable highlights in this familial case. First, the initial manifestations of PHPT in two subjects, a father and his daughter, were at the age of 15. Different from the father, whose first signs of the brown tumor were in the right maxilla, the daughter’s first signs were in the distal left radius. Second, parathyroid adenomas were observed in both subjects; the father had pathological changes in the left and right parathyroid glands, whereas the daughter had an ectopic parathyroid adenoma in the right thyroid gland. Third, it has been 28 years since the father was first diagnosed with PHPT, and we have reviewed the entire medical history from onset to recurrence. Finally, the results of genetic testing showed that both father and daughter had mutations in the CDC73 genes.This is a novel germinal mutation, c.81del, in the CDC73 gene. We considered the possibility of hyperparathyroidism-jaw tumor syndrome (HPT-JT).

## Discussion

We reported a PHPT familial case with two subjects, a father and a daughter, and both of them had suffered from the brown tumor.Genetic testing confirmed the presence of mutations in the CDC73 gene in both cases. The CDC73 gene, located on chromosome 1q31.2, parafibromin is the translated product of the CDC73 gene. This protein contributes to inhibition of cell proliferation and regulation of cell growth [7]. Inactivating the CDC73 tumor suppressor gene (previously HRPT2) increases the susceptibility of heterozygous carriers to a range of conditions, including hyperparathyroidism-jaw (HPT-JT) syndrome, familial isolated hyperparathyroidism (FIHP), and parathyroid carcinoma (PC). It has been reported that the incidence of PHPT in CDC73-related disorders can reach up to 80–95% [8]. HPT-JT syndrome is a rare autosomal dominant disorder characterised by the occurrence of hyperparathyroidism at an early age with a high incidence of parathyroid carcinomas, atypical adenomas, and ossifying fibromas of the jaw. In addition, in some rare cases, brown tumors may occur elsewhere [9], and there may be the presence of renal lesions and uterine tumors [10]. According to the literature, germline inactivating mutations of the CDC73 gene have been observed in approximately 50–75% of HPT-JT cases and 14% of FIHP [11]. Both patients in this case study exhibited mutations in the CDC 73 gene. Specifically, the father was diagnosed with a jawbone tumor and a right kidney stone, whereas the daughter, apart from being afflicted with a brown tumor in her left radius, demonstrated normal results in her kidney and uterus examinations. Therefore, we consider these 2 cases to be consistent with the presentation of HPT-JT syndrome.

PHPT generally arises from parathyroid adenoma. The parathyroid glands are located adjacent to the thyroid glands; however, approximately 2% of the population are ectopically situated [12]. As far as we know, there have been only two cases of adolescents diagnosed with ectopic parathyroid adenoma and brown tumor, as reported in previous studies [13, 14]. However, this familial case of PHPT is, to our knowledge, the first case of co-occurrence of brown tumors arising from PHPT in both father and daughter.Wang et al. [13] reported an 18-year-old male patient exhibiting a round, radiolucent, and osteolytic bone-expanding lesion in the right humeral head. The patient was diagnosed with a brown tumor, and an ectopic parathyroid adenoma was observed in the left lower lobe thyroid. Dhiwakar et al. [14] described a 17-year-old girl diagnosed with a brown tumor in both the maxilla and mandible and an ectopic parathyroid adenoma in the left thyroid gland. Coincidentally, these above-mentioned two patients and Case 2 in our report were both ectopic parathyroid adenoma in the thyroid gland. All three patients were admitted to the hospital because of pain. The initial symptom observed in each patient was attributed to the brown tumor in the corresponding part, and subsequent examinations revealed hyperparathyroidism. Ultimately, ectopic parathyroid adenoma was identified.

Given the complexity of the diagnosis of this kind of disease, clinicians should not only have a comprehensive understanding of the physiological and pathological characteristics of PHPT, but also be familiar with accurate examination schemes. Ultrasonography has become indispensable in the diagnostic process of parathyroid disease. Its sensitivity in the identification of parathyroid disease ranges from 72 to 89% [15]. A previous study found that around 62–87% of individuals possessed four parathyroid glands [16]. Parathyroid glands develop from the third and fourth pharyngeal pouches and descend with the thymus to the neck [17], and developmental anomalies may result in an ectopic parathyroid gland. Typically, normal parathyroid glands appear as hyperechoic structures. If abnormal lesions are present, they are often mixed with calcifications, thereby rendering a low or heterogeneous echo. In our study, where ultrasonography was employed in the examination, the results revealed the absence of any masses in the parathyroid glands of Case 2, although a nodule with clear boundaries was detected in the thyroid parenchyma. After reviewing clinical symptoms and laboratory results, we considered the possibility of an ectopic parathyroid tumor. Because the CT scan revealed no abnormality in the thyroid, we finally confirmed the diagnosis with the utilisation of ^99m^Tc-MIBI scintigraphy, whose sensitivity was 68–86% [18].

This lesion is caused by overactive parathyroid glands, which stimulates the activity of osteoclasts, and it is characterised by a mixture of osteoclasts and fibrous tissue within poorly mineralised woven bone. For brown tumors, the discolouration is the result of hemosiderin deposition [19]. Moreover, the imaging findings were inconclusive. It is worth mentioning that Case 2 in our research suffered from left upper limb pain due to overexertion, and PHPT was not considered a potential cause at that time. The CT examination suggested a left radial tumor, but the possibility of brown tumor was not initially considered, making it even less likely to be caused by PHPT. When the results of laboratory tests revealed elevated serum calcium and PTH levels, and the results of ultrasonography and ^99m^Tc-MIBI scintigraphy revealed an abnormal nodule in the parathyroid gland, together with enquiries about family history, the final diagnosis was reached. Brown tumor is usually accompanied by high alkaline phosphatase (over 1000 IU/L), indicating the high level of bone metabolism [20]. According to the findings of our study, the ALP level of Case 2 reached a significant value of 1892 IU/L. Therefore, it is suggested that the abnormal increase in ALP levels is often caused by bone metabolic diseases.

Parathyroidectomy is generally advised for all symptomatic PHPT patients and may also be suitable for most asymptomatic patients [21]. One study showed that patients with PHPT experienced a notable enhancement in bone density after parathyroidectomy, whereas those who did not have surgical intervention remained stable or declined in bone density [22]. In the literature review pertaining to adolescent PHPT, following the successful resection of hyperparathyroidism, the parathyroid hormone level can be reduced by more than 50% [23], but patients were prone to hypocalcemia, rapid decline of calcium level and/or spasm. In our report, hypocalcemia was observed in Case 2 one day after the operation and was rectified by taking vitamin D orally. In the follow-up duration of one to three months, the blood calcium remained below the optimal range, and the PTH level remained high until they returned to the normal level six months after operation. Therefore, it is necessary to closely monitor the dynamic changes in blood calcium. Reséndiz-Colosia et al. [24] reported that a total of 22 patients with brown tumors (68.2% in the mandible and 31.8% in the maxilla) presented a spontaneous progressive regression after parathyroidectomy. In fact, in 18 cases, the situation was resolved after a duration of ten months. Our report shows that three months after the ablation therapy, the brown tumor in the maxilla of Case 1 was mineralised.

According to the guidelines, we suggest that serum 25- hydroxycholecalciferol should be supplemented with vitamin D if the level is lower than 20 ng/mL [25]. Familial cases are more commonly caused by polygenic diseases, which have a higher and earlier recurrence rate due to the pathobiology of the disease [26], thus require more structured follow-up and monitoring. In our report, Case 1 experienced a recurrence of the condition 18 years subsequent to the initial surgery. For the following 10 years during the 28-year-long illness, there was a consistent state of normalcy. Case 2 showed normal results in the 2-year follow-up duration, with no observable indications of recurrence.

## Conclusion

The case study was conducted to document a rare familial case of PHPT arising from parathyroid adenoma with brown tumors in both a father and his daughter, and genetic testing revealed CDC73 gene mutations in both. Therefore, in the diagnostic and differential process of young patients having bone disease, clinicians should not only focus on the clinical manifestations of the skeleton, but also implement a comprehensive analysis of systemic symptoms, considering the possibility that the patient has familial PHPT.

## Data Availability

Not applicable.
